# Effects of transport stress on the oxidative index, apoptosis and autophagy in the small intestine of caprine

**DOI:** 10.1186/s12917-023-03670-9

**Published:** 2023-08-09

**Authors:** Ruini Peng, Fan Gao, Yunhai Hu, Kangli Li, Ben Liu, Wenya Zheng, Xue Yang, Wei Hu, Lucheng Zheng, Qingcan Fan, Manxin Fang

**Affiliations:** 1 College of Life Science and Resources and Environment, Yichun University, Yichun, 336000 Jiangxi China; 2 Jiangxi Lvke Agriculture and Animal Husbandry Technology Co. LTD, Yichun, 336000 Jiangxi China; 3 Engineering Technology Research Center of Jiangxi Universities and Colleges for Selenium Agriculture, Yichun, 336000 Jiangxi China

**Keywords:** Goat, Oxidative stress index, Bcl-2, Bax, PINK1, Parkin, Transport stress

## Abstract

**Background:**

Introducing new goat breeds or transferring adult goats from farms to slaughterhouses requires transportation, which can engender adverse effects, such as oxidative stress, pathological cell apoptosis and autophagy. Current evidence suggests that malondialdehyde (MDA) is a metabolite of lipid peroxidation during oxidative stress, while superoxide dismutase (SOD) and catalase (CAT) can alleviate injury caused by free radicals and reactive oxygen species (ROS). Meanwhile, Bcl-2, Bax, LC3B, PINK1 and Parkin are important proteins that participate in pathological cell apoptosis and autophagy. This study aimed to investigate the effects of transportation stress on oxidative stress indexes and expressions of Bcl-2, Bax, LC3B, PINK1 and Parkin in the small intestine of goats. Twelve healthy adult male goats from western Jiangxi province were randomly divided into control, 2 h transportation stress, and 6 h transportation stress groups (*n* = 4 per group).

**Results:**

Our results showed that MDA in the small intestine significantly increased after transportation, while SOD and CAT activities decreased, with a significantly increased apoptosis rate of the small intestine cells. The jejunum and duodenum exhibited the highest apoptosis rate in the 2 h and 6 h transportation groups, respectively. The expression of apoptosis-related genes Bcl-2 and Bax and their corresponding proteins exhibited varying degrees of down-regulation or up-regulation, while Bcl-2 and Bax genes in the small intestine were upregulated in the 6 h transportation group. In addition, autophagosomes and autophagolysosomes were found in various parts of the small intestine by transmission electron microscopy, and autophagy-related genes LC3B, PINK1 and Parkin were significantly down-regulated in the 2 h group and up-regulated in the 6 h group.

**Conclusions:**

Our results indicate that the contents of MDA, SOD and CAT in the small intestine, the expression of pathologic apoptosis-related genes Bcl-2 and Bax, and autophagy-related genes LC3B, PINK1 and Parkin correlated with stress duration caused by transportation. Moreover, this study provides a foothold for further studies on the mechanism of transportation stress in goats and improving animal welfare.

**Supplementary Information:**

The online version contains supplementary material available at 10.1186/s12917-023-03670-9.

## Background

The small intestine is a part of the gastrointestinal system that secretes various digestive juices for digesting and processing chyme. It is an important organ for animals to absorb nutrients and optimal growth and health [1]. Meanwhile, the duodenum, jejunum and ileum, which can perform different physiological functions, and any sudden change in the external environment can affect their physiological functions [2].

The transportation process is reportedly a source of stress for livestock and poultry, leading to economic losses to a certain extent [3]. During transportation, animals are affected by various factors, such as temperature, humidity, and mixing with different animals, which reduce disease resistance and even cause animal death in serious cases [4, 5]. Meanwhile, these stimuli may cause systemic changes in animals, including alterations in blood composition, hormones, enzyme activities and other metabolic processes, leading to changes in meat quality [6], increasing susceptibility to tissue and organ damage, especially to the small intestine, spleen and liver [7]. In addition, transport stress dysregulates the balance of reactive oxygen species (ROS) levels in the body and induces the accumulation of various harmful metabolites, such as malondialdehyde (MDA) and other products, while high ROS levels cause serious damage to proteins, DNA and lipids [8, 9]. The contents of superoxide dismutase (SOD) and catalase (CAT) are also subject to significant changes and maintain the body's anti-injury and anti-oxidative stress responses [10, 11]. It has been reported that transportation stress can increase MDA content in the small intestine of broilers and decrease the activities of SOD, CAT and other antioxidant enzymes, leading to oxidative stress response exacerbation [12]. Oxidative stress further induces apoptosis and autophagy reactions. Importantly, autophagy regulates the turnover of organelles and proteins in cells through targeted degradation, while apoptosis regulates cell circulation and turnover, playing an important role in response to pathogen infection and regulating normal body functions [13, 14].

Current evidence suggests that Bcl-2 and Bax genes, LC3B, PINK1 and Parkin genes play an important role in mediating cell apoptosis and autophagy [15]. Therefore, by studying the above genes and proteins, we sought to understand the relationship between autophagy and apoptosis caused by transportation stress in animals and provide novel insights into improving the meat quality of economic animals and animal welfare by reducing stress levels.

## Results

### Effect of transport stress on small intestinal oxidation index

**Fig. 1:**
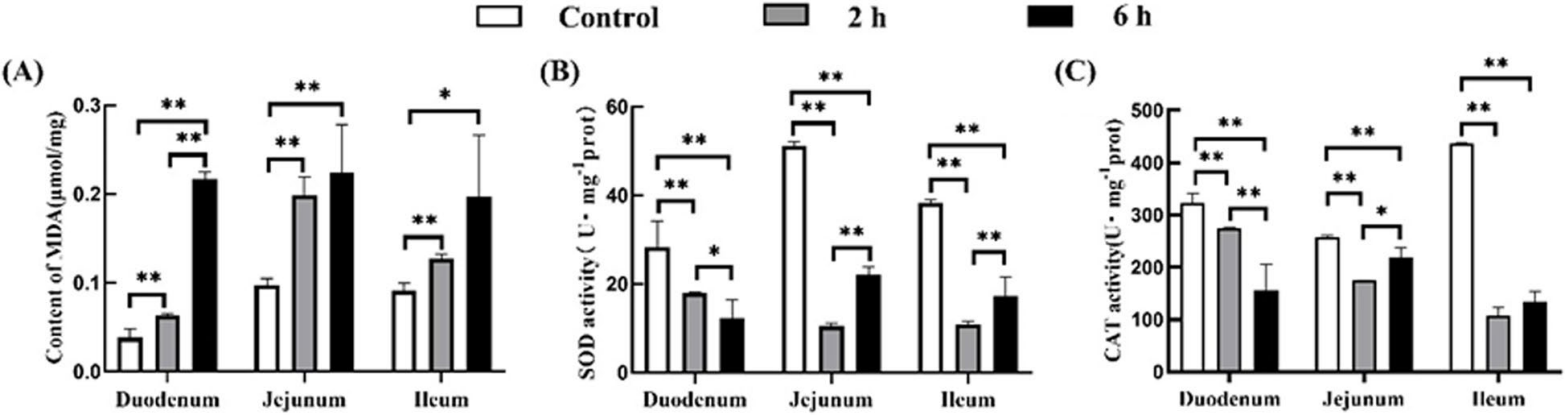
Effect of transportation stress on goats’ biochemical indexes MDA, SOD and CAT. **A** MDA level; **B** SOD activity; **C** CAT activity. Data are presented as the means ± SD; *: significant difference (*P* < 0.05); **: Highly significant difference (*P* < 0.01)

Duodenum, jejunum, and ileum samples of the control and transportation treatment groups were collected to quantify MDA content, SOD and CAT activities (Fig. 1A). Compared with the control group, MDA content in the duodenum, jejunum and ileum of the 2 h transportation group (*P*_s_ < 0.01), and MDA content in the duodenum and jejunum in the 6 h transportation group significantly increased (*P* < 0.01, *P* < 0.01, and *P* < 0.05, respectively); MDA content in the duodenum of the 6 h transportation group was significantly higher than the 2 h transportation group (*P* < 0.01). As shown in Fig. 1B, compared with the control group, SOD activity in the duodenum, jejunum and ileum in the 2 h and 6 h transportation groups significantly decreased (*P* < 0.01), and SOD activity in the duodenum, jejunum and ileum of 6 h transportation group was significantly lower than the 2 h transportation group (*P* < 0.05, *P* < 0.01, and *P* < 0.01, respectively). Compared with the control group, CAT activity in the duodenum, jejunum and ileum in the 2 h and 6 h transportation groups were significantly decreased (*P* < 0.01), and CAT activity in the duodenum and jejunum in the 6 h transportation group was significantly lower (*P* < 0.01) and higher (*P* < 0.05) than the 2 h transportation group, as shown in Fig. 1C.

### Effects of transport stress on apoptosis and expression of Bcl-2 and Bax in caprine small intestine

#### Apoptosis of small intestinal cells before and after transportation

**Fig. 2:**
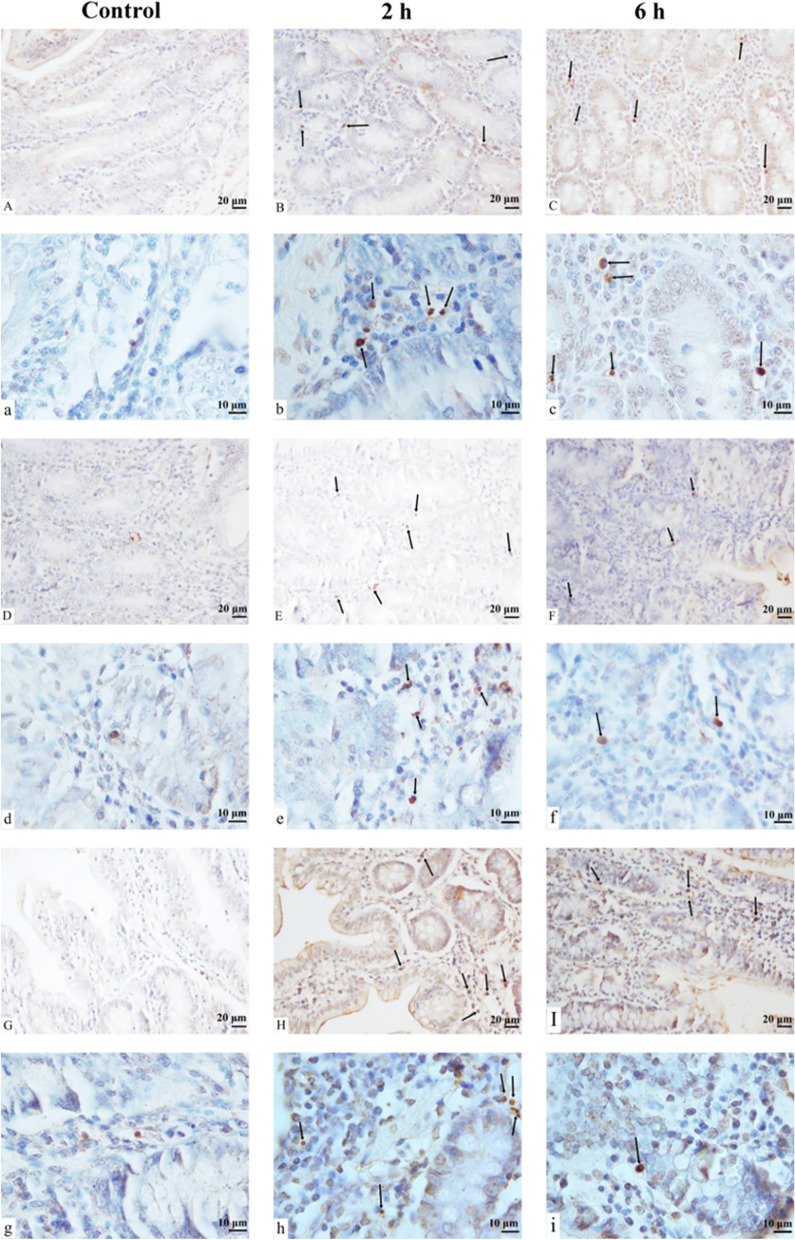
TUNEL staining showing the apoptosis of the small intestines of goats in the control group, 2 h transport group, and 6 h transport group. Scale bar 10–20 μm. A(a) ~ C(c) Apoptosis of the duodenum. D(d) ~ F(f) Apoptosis of the jejunum. G(g) ~ I(i) Apoptosis of the ileum. Arrows: Terminal deoxynucleotidyl transferase-mediated dUTP nick end labeling (TUNEL) positive cells

**Fig. 3:**
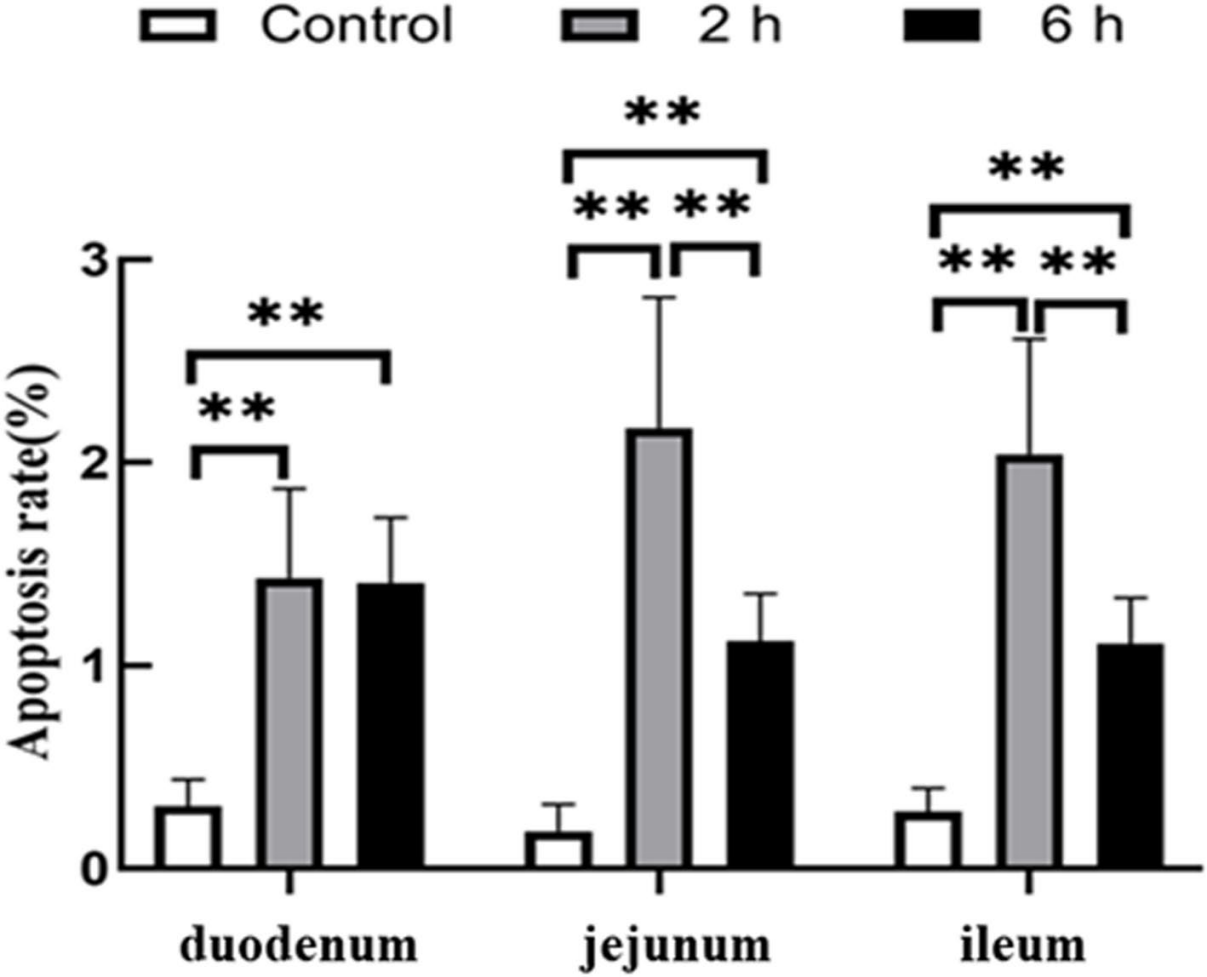
The TUNEL apoptosis detection showing apoptosis rate of small intestinal cells from the control group, 2 h transport group, and 6 h transport group. Data are presented as the means ± SD; **: Highly significant difference (*P* < 0.01)

The TUNEL apoptosis detection kit was used to evaluate intestinal cell apoptosis. The nuclei of apoptotic cells were stained brown after TUNEL staining and were aggregated and separated from the surrounding tissues (Fig. 2). The apoptotic cells were mainly located in the lamina propria and some in the mucosa, while the normal nuclei were stained blue. After transportation, the number of apoptotic cells in the intestinal tract of goats was altered. The apoptosis rates in the duodenum, jejunum and ileum in 2 h and 6 h transportation groups were significantly higher than in the control group (*P* < 0.01), and the apoptosis rate in the jejunum and ileum in the 6 h group was significantly lower than in the 2 h transportation group (*P* < 0.01). However, there was no significant difference in the apoptosis rate in the duodenum between the 2 h and 6 h groups (*P* > 0.05) (Fig. 3). The number of small intestinal apoptotic cells and the apoptosis rate increased following transportation, indicating that transportation stress could induce apoptosis of small intestinal cells in goats.

#### Expression of Bax and Bcl-2 genes in the small intestine of goats

**Fig. 4:**
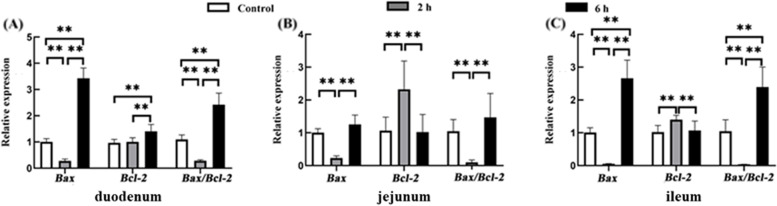
The relative expression of Bax and Bcl-2 genes in the small intestine from the control group, 2 h transport group, and 6 h transport group by qRT-PCR. Reference gene: β-actin; *n* = 4; Data are presented as the means ± SD; **: Highly significant difference (*P* < 0.01)

Intestinal Bax and Bcl-2 genes of goats in different transport groups were quantified by qRT-PCR. The results showed that compared with the control group, Bax gene expression in the duodenum of the 2 h and 6 h transportation groups was significantly decreased (*P* < 0.01) and increased (*P* < 0.01), respectively. Bcl-2 gene expression in the duodenum of the 2 h and 6 h transportation groups was comparable (*P* > 0.05) and significantly increased (*P* < 0.01), respectively, compared to the control group. Bax/Bcl-2 significantly decreased (*P* < 0.01) and increased (*P* < 0.01) in the 2 h and 6 h transportation groups. Moreover, Bax, Bcl-2, and Bax/Bcl-2 levels in the 6 h group were significantly higher than in the 2 h group (*P* < 0.01). Bax and Bax/Bcl-2 expression in the jejunum were significantly lower in the 2 h transportation group than in the control group (*P* < 0.01), while bcl-2 gene expression was higher (*P* < 0.01). Bax, Bcl-2, and Bax/Bcl-2 levels in the 6 h group were comparable with the control group (*P* > 0.05). Bax and Bax/Bcl-2 in the 6 h transportation group were significantly higher than in the 2 h group (*P* < 0.01). Bax and Bax/Bcl-2 in the ileum were significantly lower in the 2 h group, and bcl-2 higher (*P* < 0.01) than in the control group (*P* < 0.01). Compared with the control group, Bax and Bax/Bcl-2 were significantly increased in the 6 h group (*P* < 0.01), while bcl-2 had no significant difference (*P* > 0.05). Bax and Bax/Bcl-2 expression in the 6 h group was significantly higher than in the 2 h group (*P* < 0.01), while bcl-2 was significantly lower (*P* < 0.01) (Fig. 4).

#### Expression of Bax and Bcl-2 proteins in the small intestine of goats

**Fig. 5:**
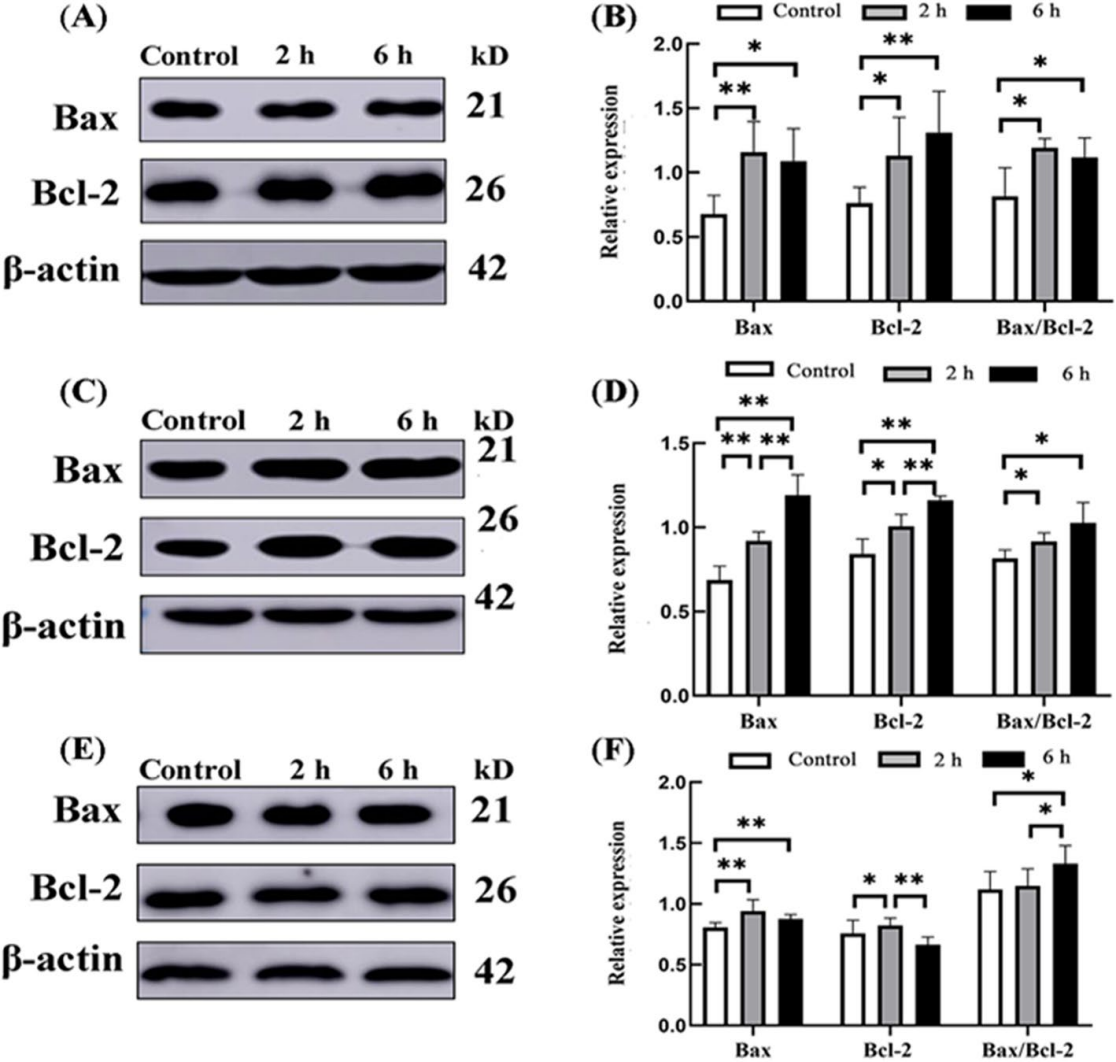
The relative protein expression of Bax and Bcl-2 in the small intestine of goat from the control group, 2 h transport group, and 6 h transport group. **A**, **B** Western blot and quantitative analysis of duodenum Bax and Bcl-2 protein, respectively. **C**, **D** Western blot and quantitative analysis of jejunum Bax and Bcl-2 protein, respectively. **E**, **F** Western blot and quantitative analysis of ileum Bax and Bcl-2 protein, respectively. Reference protein: β-actin; *n* = 4; Data are presented as the means ± SD; *: Significant difference (*P* < 0.05); **: Highly significant difference (*P* < 0.01)

The Bax and Bcl-2 proteins in the small intestine of goats from different transportation groups were detected by Western blot. The results showed that, compared with the control group, Bax expression in the duodenum was significantly higher in the 2 h (*P* < 0.01) and 6 h (*P* < 0.05) groups. Similar findings were found for Bcl-2 (*P* < 0.05, *P* < 0.01) and Bax/Bcl-2 (*P*_s_ < 0.05) expression. No significant differences in Bax, Bcl-2 and Bax/Bcl-2 were found between the 6 h group and 2 h group (*P*_s_ > 0.05). In the jejunum, Bax was significantly increased in the 2 h and 6 h transportation groups (*P* < 0.01), compared with the control group. Nevertheless, in the 2 h and 6 h groups, bcl-2 (*P* < 0.05, *P* < 0.01) and Bax/Bcl-2 (*P*_s_ < 0.05) were significantly upregulated; Bax and Bcl-2 expression in the 6 h group was significantly higher than in the 2 h group (*P* < 0.01), while the difference in Bax/Bcl-2 expression was not significant (*P* > 0.05). In the ileum, compared with the control group, Bax was significantly increased in the 2 h and 6 h transportation groups (*P*_s_ < 0.01), while bcl-2 was increased in the 2 h transportation groups (*P* < 0.05) and no significant difference was found with the 6 h transportation groups (*P* > 0.05). In contrast with the control group, no significant difference in Bax/Bcl-2 levels was found in the 2 h transportation group (*P* > 0.05), while a significant increase was observed in the 6 h transportation group (*P* < 0.05). Finally, compared with the 2 h group, Bax in the 6 h group was comparable (*P* > 0.05), while Bcl-2 and Bax/Bcl-2 expression was significantly decreased (*P* < 0.01) and increased (*P* < 0.05), respectively (Fig. 5).

#### Localization and expression of Bax and Bcl-2 proteins in the small intestine of goats

**Fig. 6:**
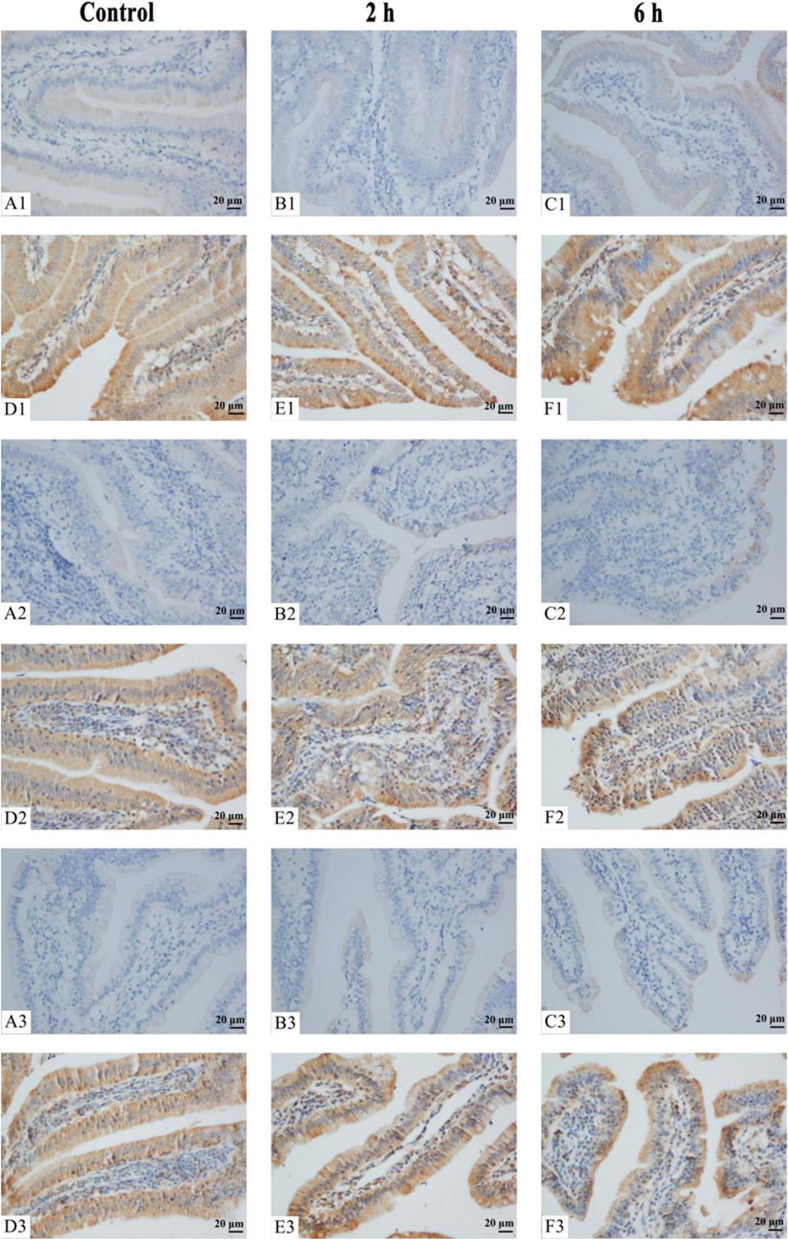
Immunohistochemical staining showing the protein expression of Bax and Bcl-2 in the small intestines of goats in the control group, 2 h transportation group and 6 h transportation group. Scale bar 20 μm. A1 ~ C1 The expression of Bcl-2 in the duodenum. D1 ~ F1: The expression of Bax in the duodenum. A2 ~ C2 The expression of Bcl-2 in the jejunum. D2 ~ F2 The expression of Bax in the jejunum. A3 ~ C3 The expression of Bcl-2 in the ileum. D3 ~ F3 The expression of Bax in the ileum

Immunohistochemistry was used to detect the distribution and localization of Bax and Bcl-2 proteins in the small intestine of goats. As shown in Fig. 5, Bax and Bcl-2 proteins were upregulated in the small intestine of goats before and after transportation, and their distribution and location did not change significantly. Bcl-2 was mainly expressed in intestinal mucosal epithelial cells. Bax was expressed in both mucosa and submucosa; the columnar cells of the mucosal epithelium, intestinal glandular cells of the small intestinal glands, and lamina propria cells showed strong positive expression, unlike goblet cells (Fig. 6).

### Effects of transport stress on autophagy and PINK1/Parkin expression in small intestinal cells

#### Observation of pathological changes in the small intestinal ultrastructure in goats

**Fig. 7:**
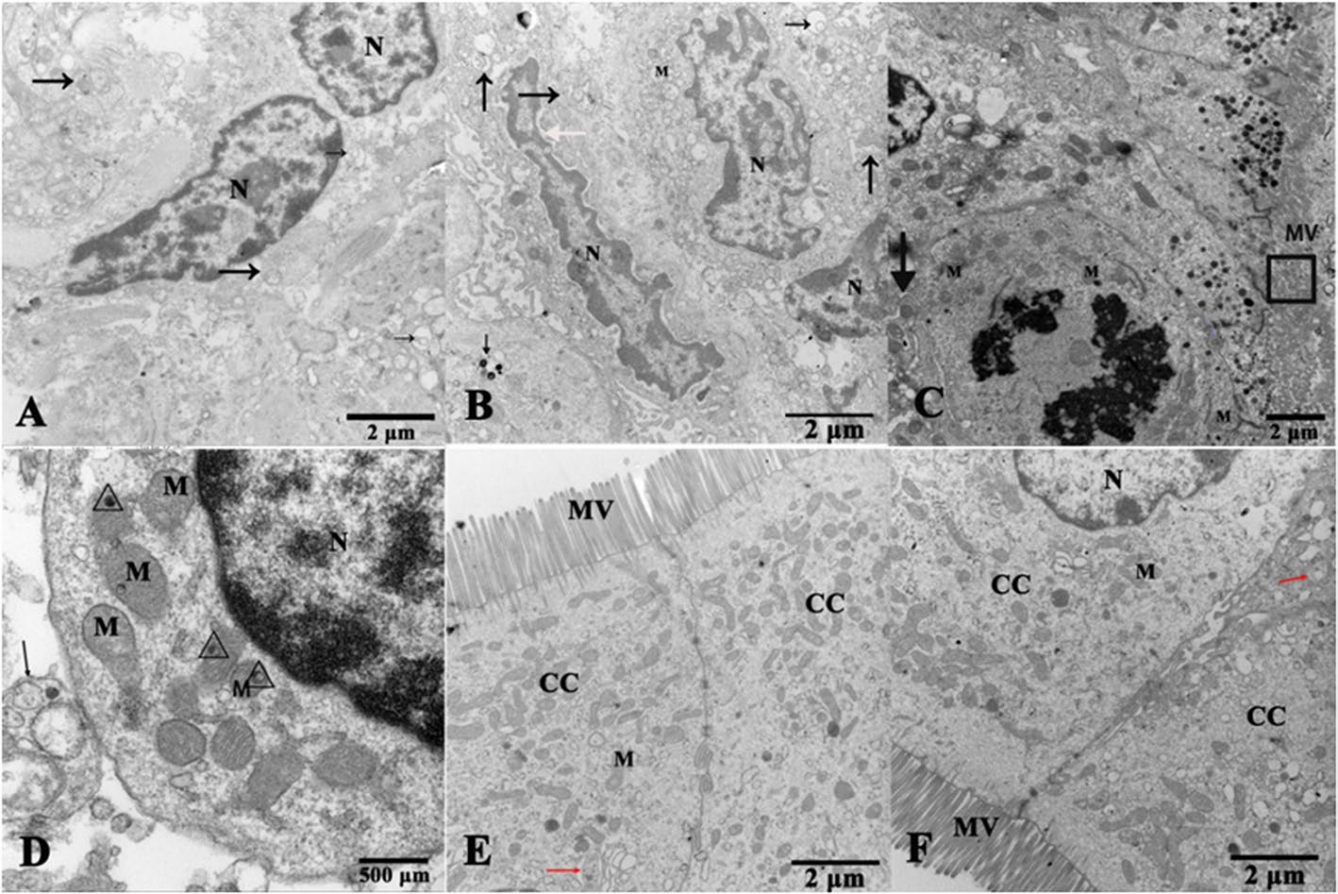
Transmission electron microscope show ultrastructure of the small intestines of goats from the transportation group. **A**, **B** Ultrapathological changes of the duodenum. **C**, **D** Ultrapathological changes of jejunum. **E**, **F** Ultrapathological changes of the ileum. N: Nucleus; M: Mitochondria; CC: Ciliated cells; MV: Microvilli; Rectangular box: The microvilli were bent and prostrate; Triangular box: Flocculent material; Black arrows: Autophagic vesicles with partially degraded electron-dense cytoplasm; Red arrow: Autophagosome; Green arrow: The nuclear membrane was ruptured, and chromatin was fragmented; White arrow: The spacing of the nucleus were widened

The intestinal cell structure of goats was damaged under transportation stress. In terms of ultrastructure, autophagosomes and autophagolysosomes were found in the duodenum, jejunum and ileum of the transportation group, with widened nuclear space, nuclear membrane rupture, chromatin disintegrating into fragments, microvilli bent and collapsed, and mitochondria swelling with floccular appearance (Fig. 7). These results indicate that transport stress damages intestinal cells and induces the formation of autophagosomes.

#### Expression of LC3B, PINK1 and Parkin genes in the small intestine of goats

**Fig. 8:**
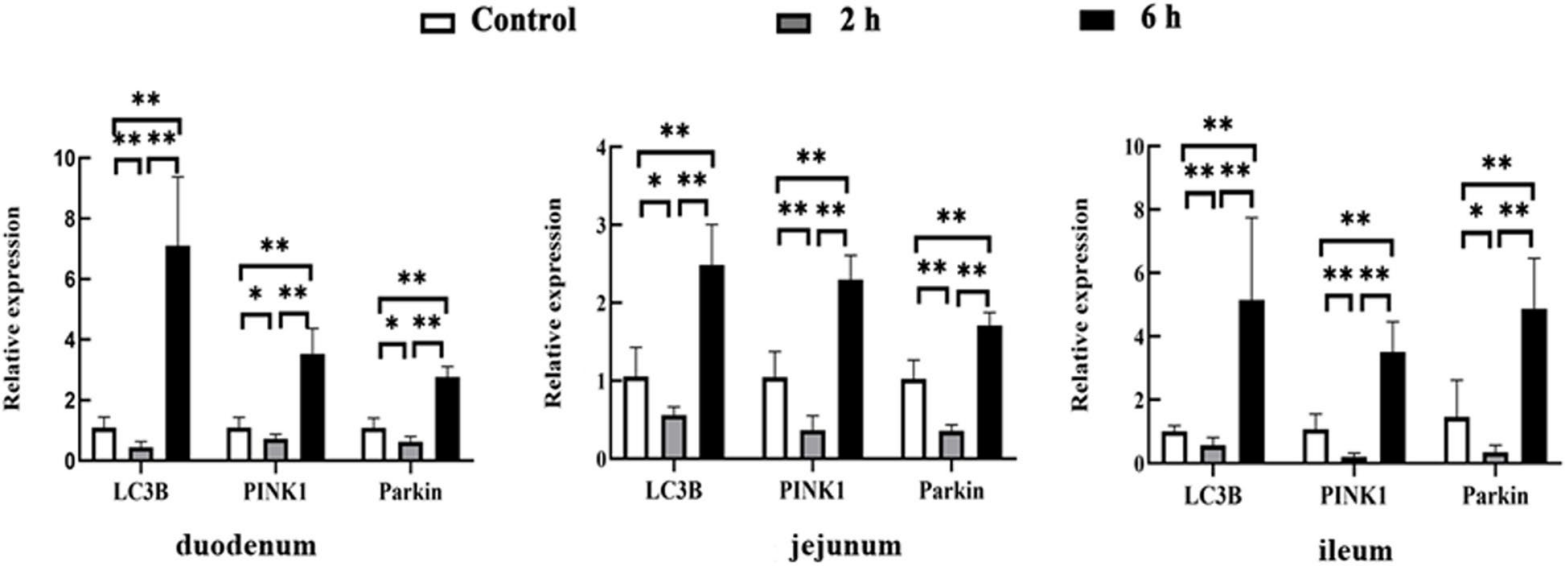
The relative expression of LC3B, PINK1 and Parkin genes in the small intestine of goats from the control group, 2 h transport group, and 6 h transport group by qRT-PCR. Reference gene: β-actin; *n* = 4; Data are presented as the means ± SD; *: Significant difference (*P* < 0.05); **: Highly significant difference (*P* < 0.01)

RNA was extracted from each part of the small intestine for reverse transcription, and the relative expression levels of LC3B, PINK1 and Parkin genes were detected by qRT-PCR. The results showed that compared with the control group, the expression levels of LC3B, PINK1 and Parkin genes in the duodenum, jejunum and ileum in the 2 h transportation group exhibited different degrees of decrease, and the expression of LC3B, PINK1 and Parkin genes in the duodenum, jejunum, and ileum from the 6 h transportation group increased significantly compared with the control group and the 2 h group (*P*_s_ < 0.01) (Fig. 8).

#### Expression of LC3B, PINK1 and Parkin proteins in the small intestine of goats

**Fig. 9:**
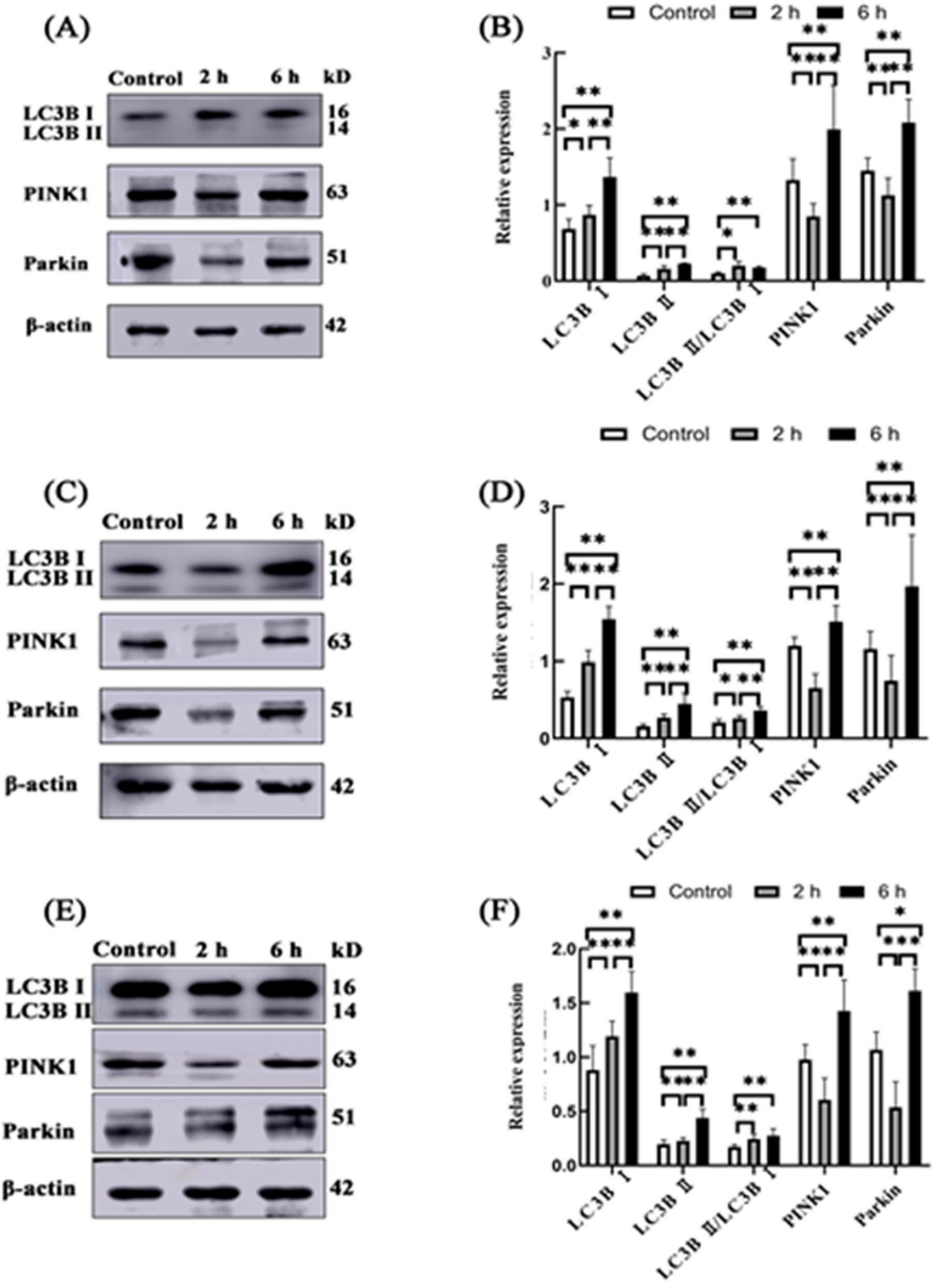
The relative protein expression of LC3B, PINK1 and Parkin in the small intestine of goats from the control group, 2 h transportation group and 6 h transportation group. **A**, **B** Western blot analysis and quantitative analysis of duodenum LC3B, PINK1 and Parkin protein, respectively. **C**, **D** Western blot and quantitative analysis of jejunum LC3B, PINK1 and Parkin protein, respectively. **E**, **F** Western blot and quantitative analysis of ileum LC3B, PINK1 and Parkin protein, respectively. Reference protein: β-actin; *n* = 4; Data are presented as the means ± SD; *: Significant difference (*P* < 0.05); **: Highly significant difference (*P* < 0.01)

The relative expression levels of LC3B, PINK1 and Parkin proteins were detected by Western blot. The results showed that in the duodenum, jejunum and ileum, protein expressions of LC3B I and LC3B II and LC3B II /LC3B I were upregulated in the 2 h and 6 h transportation groups compared with the control group, and PINK1 and Parkin were downregulated in the 2 h transportation group compared with the control group. The protein expression levels of PINK1 and Parkin in the 6 h group were higher than in the control and 2 h transportation groups (Fig. 9).

#### Localization and expression of LC3B, PINK1 and Parkin proteins in the small intestine of goats

**Fig. 10:**
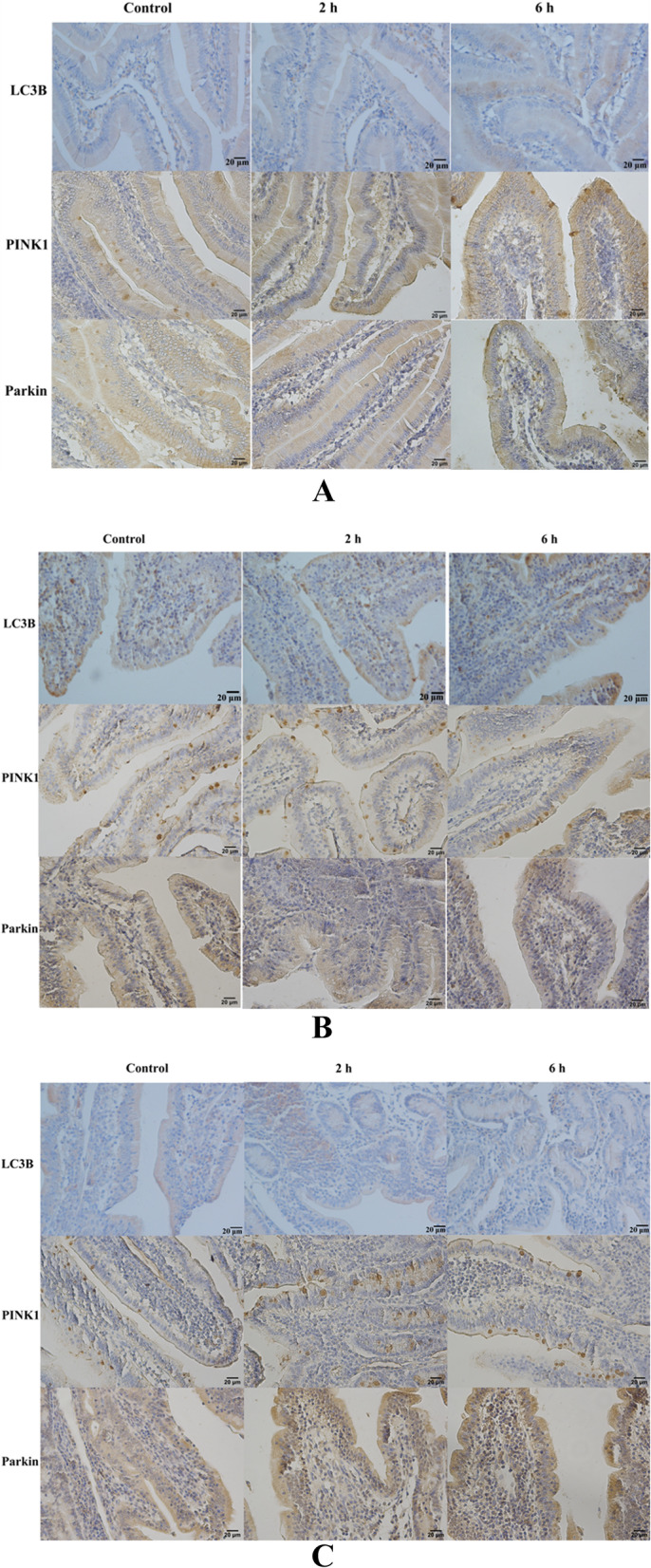
Immunohistochemical staining showing the protein expression of LC3B, PINK1 and Parkin in the small intestines of goats from the control group 2 h transportation group and 6 h transportation group. Scale bar 20 μm. **A**, **B**, **C** The expression of LC3B, PINK1 and Parkin proteins in the duodenum, jejunum and ileum, respectively

Immunohistochemical results showed that LC3B, PINK1 and Parkin were expressed in the small intestine of goats before and after transportation and were localized in the same regions. LC3B was mainly expressed in the intestinal mucosa and submucosa, and positive expression was found in intestinal epithelial cells, lamina propria cells and intestinal glandular cells. PINK1 was upregulated in mucosa, submucosa and muscular, and goblet cells in mucosa and intestinal gland exhibited a strong positive expression. Similar to PINK1, Parkin was expressed in the mucosa, submucosa and lamina propria but not in goblet cells (Fig. 10).

## Discussion

It is widely acknowledged that high temperature, high humidity and other harsh transport conditions can significantly influence the physiological state of animals in different aspects [16]. An increasing body of evidence suggests that transport stress may reduce the digestion and absorption levels of economic animals, reduce feed conversion, and lead to changes in meat quality, metabolism and absorption disorders [17, 18]. The small intestine is important for animals to absorb nutrients. Notwithstanding that the past decade has witnessed significant scientific progress, few reports have documented the molecular changes in the small intestine during transportation stress in goats [19, 20]. It has been shown that transport stress can cause significant damage to the small intestine of rats and alter the gene expression spectrum, especially in the jejunum [21].

**Fig. 11:**
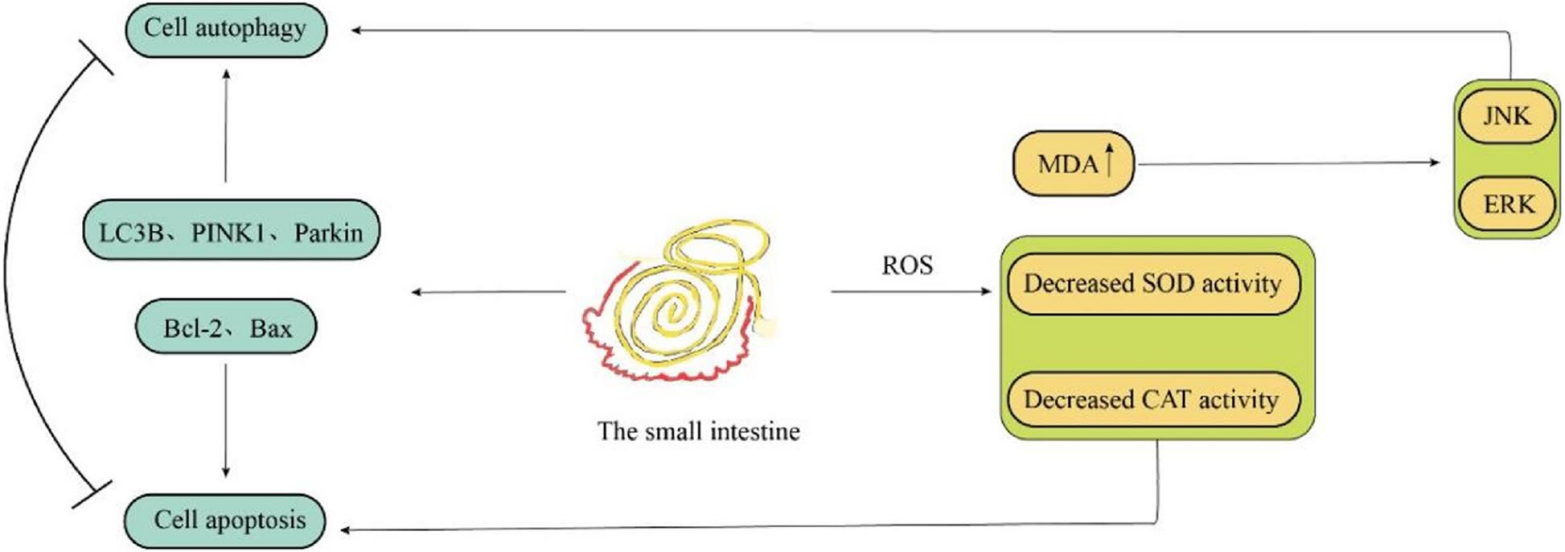
Transport stress leads to the activation of metabolic pathways

In this study, we found that transport stress could increase the oxidative stress level, apoptosis rate and autophagy level in the small intestine of goats. Furthermore, MDA content increased significantly at 2 h and 6 h, while SOD and CAT activities decreased by varying degrees. MDA is a well-recognized by-product of ROS metabolism and can be used as a marker of oxidative stress to judge oxidative stress levels, indicating that transportation stress causes oxidative damage in the small intestine of goats. In addition, high levels of ROS induce autophagy, and ROS can participate in the autophagy process by activating the mammalian target of the rapamycin (mTOR) system [22, 23]. At the same time, oxidative stress metabolites such as MDA also affect C-Jun N-terminal kinase (JNK) and extracellular regulated kinase (ECK), mediated by MDA levels. ERK affects the formation of autophagosomes (Fig. 11) [24]. By observing the ultrastructure of the small intestine, autophagosomes and autophagolysosomes were found in the duodenum, jejunum and ileum. Meanwhile, the expression of LC3B, PINK1 and Parkin genes were downregulated in the duodenum, jejunum and ileum after 2 h of transportation but upregulated at 6 h. Quantitative analysis of LC3B, PINK1 and Parkin proteins showed that the expression of LC3BI and LC3B II was higher in the 2 h transportation group than in the control group, while consistent levels were observed in the 6 h transportation group. At the same time, Bax gene expression was lower, and Bax protein was higher in the 2 h transportation group than in the control group. This finding may be due to the rapid expression of autophagy and apoptosis-related genes in the early stage of transport stress because of the drastic changes in environmental conditions to regulate the body to make appropriate feedback and coping strategies to the stress stimulus [25, 26]. Furthermore, in the 2 h and 6 h transportation groups, gene and protein expression of Bax in the duodenum and ileum exhibited opposite trends. Consistent findings were observed for Bcl-2 gene and protein expression in the jejunum. Bax gene expression was the highest in the 6 h group, with the lowest amount of protein synthesis. Bcl-2 exhibited high expression in the 2 h transportation group but low protein levels. This finding may be due to the high mRNA synthesis efficiency but low translation efficiency of the Bax gene in the duodenum and ileum of the 6 h group, while the mRNA translation efficiency of the Bcl-2 gene in the jejunum of the 6 h group was higher.

Autophagy is a highly complex regulatory process during which part of cytoplasm and organelles are isolated in unique double-or multilayered autophagosomes, which are eventually transported to lysosomes for degradation to remove damaged organelles or misfolded proteins and maintain normal physiological functions [27]. Apoptosis refers to programmed cell death, involving a variety of signal cascade reactions and the activation of a variety of decomposition enzymes, especially proteases, which rapidly destroy the cell structure and organelles and eventually lead to cell contraction, nuclear chromatin agglutination, and nuclear fragmentation [28, 29]. Therefore, apoptosis and autophagy are two processes that remove excess, damaged, or aging cells or organelles and play an important role in regulating the body's resistance to various adverse factors [30, 31]. In addition, ROS imbalance caused by transport stress and weakened activity of various antioxidant enzymes can mediate autophagy and apoptosis. Meanwhile, autophagy and apoptosis are tightly associated. Overwhelming evidence substantiates that superoxide dismutase (SOD) and catalase (CAT) can inhibit autophagy and play important anti-oxidation and anti-apoptosis roles [32]. It has been reported that when SOD content and CAT activity are significantly reduced, autophagy and apoptosis are induced, and significant autophagy can further activate apoptosis [33, 34].

## Conclusion

Overall, our study indicates that transportation stress can cause oxidative stress, apoptosis and autophagy in the duodenum, jejunum and ileum of goats. Oxidative stress is mediated by increasing the MDA content of ROS metabolites and decreasing the activities of SOD and CAT. Meanwhile, apoptosis genes Bcl-2, Bax and autophagy-related genes LC3B, PINK1 and Parkin exhibited different degrees of dysregulation in various segments of the small intestine, thus mediating the occurrence of apoptosis and autophagy reaction. These results indicate that the above stress indicators and related genes play an important role in the development of goat transport stress, and the exploration of the underlying mechanisms will be the focus of our next studies.

## Methods

### Animals and experimental design

According to the previous literature [35], twelve 1-year-old male goats without clinicopathological manifestations and with similar physiques were selected, with a body weight of 13.89 ± 2.96 kg. They were randomly divided into the Control group (*n* = 4), 2 h transportation group (*n* = 4), and 6 h transportation groups (*n* = 4). Briefly, the control group were directly transported to the abattoir (1.5 h journey) a day prior to slaughter and housed in resting pens at room temperature for 24 h (16:00 to next day 16:00) with water ad libitum before slaughter.while the transportation group goats were transported by road at a speed of 35 ~ 45 km/h with the vehicle's temperature set at 28 ~ 32 °C, and no food and water was provided during transportation. No accidents occurred during the transport period. After transportation, the goats were not allowed to rest and were euthanized while under anesthesia by i.v. injection of 90 mg/kg pentobarbital sodium. Death was confirmed by auscultation for cardiac arrest. The euthanasia method used was consistent with the recommendations by the Chinese Association for Laboratory Animal Sciences. The animal care and experimental procedures used in this study conformed to the regulations and guidelines of the regional Animal Ethics Committee and the Ethical and Animal Welfare Committee of Yichun University (License number: JXSTUDKY2019009).

### Sample collection

The duodenum, jejunum and ileum samples were harvested by sterile surgical instruments, take 4 cm from each section of bowel. The sample was rinsed several times with PBS buffer solution and drained with filter paper. One part was fixed with 4% paraformaldehyde and 2.5% glutaraldehyde and stored at 4 °C for later use. The remaining part was put into a cryopreservation tube and stored at -80 °C after quick-freezing with liquid nitrogen.

### Quantification of MDA content and SOD and CAT activity

Samples were taken from -80 °C and homogenized at 50 mg per sample. MDA (S0131, Shanghai Beyotime Biological Co., Ltd) working fluid was prepared as follows [36]: the standard solution was diluted to 1, 2, 5, 10, 20 and 50 μM for standard curve making. 0.1 mL lysate was added to the centrifuge tube as blank control, 0.1 mL of the above standard solution with different concentrations was added to make the standard curve, and 0.1 mL sample was added for determination. Subsequently, the mixture was heated at 100 °C for 15 min, cooled to room temperature and centrifuged at 1000 × g for 15 min. 200 μL of the supernatant was used to measure the absorbance at 532 nm on a 96-well plate. After the MDA content of the sample was calculated according to the standard curve, the MDA content in the sample was expressed by tissue weight.

50 mg tissue was added to 500 μL of the sample preparation solution and centrifuged to get the supernatant, which was used to determine the protein concentration. Preparation of enzyme working fluid, the reaction starts working fluid reserve. Sample holes and various blank control holes were set in the 96-well plate. The total system of each well was 200 μL, including 160 μL enzyme working solution and the remaining 40 μL according to the purpose of each well. For blank control 1, 20 μL detection buffer and 20 μL reaction starting solution were added. For blank control 2, a 40 μL detection buffer was added. Finally, for blank control 3, 20 μL test buffer and 20 μL test sample were added. 20 μL of the test sample was added to 20 μL of the working fluid and mixed homogeneously. After incubation at 37 °C for 30 min, the absorbance was measured at 450 nm. SOD activity was calculated as follows:

$$\mathrm{SOD}\;\mathrm{inhibition}\;\mathrm{rate}\left(\%\right)=\frac{({\mathrm A}_{\mathrm{blank}\;\mathrm{control}\;1}-{\mathrm A}_{\mathrm{blank}\;\mathrm{control}\;2})-({\mathrm A}_{\mathrm{determination}}-{\mathrm A}_{\mathrm{blankcontrol}\;3})}{({\mathrm A}_{\mathrm{blank}\;\mathrm{control}\;1}-{\mathrm A}_{\mathrm{blan}\;\mathrm{kcontrol}\;2})}\times100\%$$

$$\mathrm{SOD}\;\mathrm{activity}\;\mathrm{unit}=\frac{\mathrm{SOD}\;\mathrm{inhibition}\;\mathrm{rate}}{(1-\mathrm{SOD}\;\mathrm{inhibition}\;\mathrm{rate})}\mathrm{units}$$

The unit of SOD activity was converted to U/mg protein according to the protein concentration and dilution ratio of the sample.

The tissue was lysed with the proper amount of lysate, and the protein concentration of the sample was determined. For determination of the standard curve: 0, 12.5, 25, 50, and 75 μL of 5 mM hydrogen peroxide solution was added to the centrifuge tube, and catalase detection buffer was added to obtain a final volume of 100 μL. 4 μL of each solution was taken from the 96-well plate, added to 200 μL of color working solution, and incubated at 25 °C for 15 min. Then, the absorbance was measured at 520 nm. For sample determination, the sample was put into a centrifuge tube and catalase detection buffer was added until 40 μL of the sample was obtained. For the blank control, 40 μL catalase detection buffer was directly added to 10 μL of 250 mM hydrogen peroxide solution and allowed to react at 25 °C for 5 min. 10 μL of the reaction system was added to the centrifuge tube with 40 μL catalase0 detection buffer within 15 min after termination of the reaction. After mixing, 10 μL was added to the 96-well plate, and 200 μL of chromosome working solution was added for incubation at 25 °C for 15 min. The absorbance at 520 nm was measured. The catalase activity in the sample was calculated as follows:

$$\mathrm{CAT}\;\mathrm{enzyme}\;\mathrm{activity}=\frac{\mathrm{micromoles}\;\mathrm{of}\;\mathrm{hydrogen}\;\mathrm{peroxide}\;\mathrm{consumed}\times\mathrm{dilution}\;\mathrm{ratio}}{\mathrm{number}\;\mathrm{of}\;\mathrm{reaction}\;\mathrm{minutes}\;\times\;\mathrm{sample}\;\mathrm{volume}\;\times\;\mathrm{protein}\;\mathrm{concentration}}$$

### Paraffin sections preparation

The tissue samples fixed in 4% paraformaldehyde were rinsed with running water, followed by dehydration in different concentrations of alcohol and soaking in xylene and paraffin, and tissues were embedded in liquid paraffin. Following solidification, paraffin-embedded tissue samples were cut into 5 μm-thick sections using a rotary microtome (RM2235, Leica Biosystems, Buffalo Grove, IL, USA). Finally, all paraffin sections were stored at 4 °C for further research.

### TUNEL cell apoptosis was detected

The small intestine samples were fixed with 4% paraformaldehyde overnight and washed with water. After paraffin embedding, 5 μm-thick sections were obtained. Paraffin sections were routinely dewaxed, hydrated and soaked in distilled water and PBS for 5 min each. The tissues were treated with a working solution of protease K (15 μg/mL, pH 7.4) at 37 °C for 20 min and washed with PBS 5 times for 5 min each time. 50 μL of TUNEL reaction mixture (TdT: Fluorescein labeled dUTP = 1:9) was added, while 50 μL of fluorescein-labeled dUTP was added to the negative control group, and the reaction was conducted in a dark wet box at 37 °C for 1 h. The tissues were washed with PBS 3 times (5 min/time). 50 μL transforming agent POD (converter-POD). The mixture was allowed to react in the dark wet box at 37 °C for 30 min. After washing with PBS 3 times, 50 μL DAB substrate was added, and a color reaction was performed at room temperature. Conventional dehydration, sectioning and hematoxylin staining were conducted, and images were observed under a microscope and photographed. Microscope observation and photography. The results of cell apoptosis were analyzed, and the apoptosis rate was calculated using the following formula:

$$\mathrm{Apoptosis}\;\mathrm{rate}=\frac{\mathrm{Apoptotic}\;\mathrm{cells}}{\mathrm{Total}\;\mathrm{cell}}\times100\%$$

### Immunohistochemistry

The paraffin sections (5 μm-thick) were deparaffinized in xylene, rehydrated with graded ethanols, and washed with water. Antigen retrieval was performed by microwaving the sections in 10 mM sodium citrate buffer (pH 6.0) and cooling them to room temperature. The endogenous horseradish peroxidase activity was inhibited with 3% H_2_O_2_ for 15 min. After the nonspecific binding was blocked with 10% horse serum at 37 °C for 1 h, the sections were incubated with primary antibody at 4 °C overnight. The primary antibodies used in this study include the mouse anti-Bcl-2 antibody (1:20; sc-7382, Santa Cruz Biotechnology (Shanghai) Co., LTD. China), and the mouse anti-Bax antibody (1:500; ab77566, Ai Bo Kang (Shanghai) Trading Co., LTD, China), and the mouse anti-LC3B antibody (1:200; ab229327, Ai Bo Kang (Shanghai) Trading Co., LTD, China), and the mouse anti-PINK (ab137361, Ai Bo Kang (Shanghai) Trading Co., LTD, China), and the mouse anti-Parkin (bs-23687R, Beijing Boaosen Biotechnology Co. LTD, China). The sections were then incubated with a biotin-labeled goat anti-mouse IgG antibody (1:1000; ab6789, Abcam) and a streptavidin-conjugated HRP complex (SP9002, Zhongshan Golden Bridge, Beijing, P. R. China). Finally, the signals were visualized with the DAB Horseradish Peroxidase Color Development Kit (ZLI9018, Zhongshan Golden Bridge) and counterstained with hematoxylin.

### Total RNA extraction and reverse transcription

25 mg of the small intestine samples were put into a 1.5 mL centrifuge tube, and 600 μL RNAiso Plus was added for ice homogenization. After homogenization, the samples were placed on ice for further lysis for 5 min and centrifuged at 13,600 × g at 4 °C for 5 min. The uncleaved tissue was deposited at the bottom of the tube. 500 μL of the upper liquid was transferred to a new 1.5 mL centrifuge tube. 1/5 of the homogenate lysed by chloroform (100 μL) was added and shaken vigorously 60–80 times until the solution turned milky white. After the mixture was allowed to stand for 2 min, centrifugation was conducted at 13,600 × g for 15 min at 4 °C. 260–280 μL of the upper liquid was placed into a new 1.5 mL centrifuge tube, and isopropyl alcohol was added and mixed thoroughly. The mixture was allowed to stand at room temperature for 10 min and centrifuged at 16,000 × g at 4 °C for 10 min. The supernatant was discarded, and the precipitate was retained; 750 μL 75% ethanol was added, followed by centrifugation as described above. The RNA deposit was allowed to dry at room temperature. After precipitation and drying, it was dissolved in 20 μL DEPC water. The concentration of the RNA solution was determined. When the concentration was less than 500 ng/μL, 20 μL was transferred into a new 1.5 mL centrifuge tube. Digestion of the mixed genomic DNA in the RNA solution in the previous step was conducted using the following:

**Table:** 

Reagent	Volume
10 × Reaction Buffer	3 μL
Recombinant DNase I	3 μL
DTT (DEPC-treated water made up to 50 mM)	0.5 μL
Rnase Inhibitor (40 U/μL)	0.5 μL
RNA liquid + DEPC water	23 μL

After mixing, the mixture was digested at 37 °C for 30 min. 270 μL DEPC water, 150 μL of each saturated phenol solution and chloroform were added to the digested solution, shaken vigorously, and allowed to stand at room temperature for 2 min, followed by centrifugation at 13 600 × g for 15 min at 4 °C. 320 μL of the upper layer solution was added to a new 1.5 mL centrifuge tube, followed by 890 μL cold ethanol (precooled at -20 °C) and 35.5 μL 3 M sodium acetate (NaAc), mixed, and overnight at -80 °C. On the second day, the RNA solution that was deposited overnight was centrifuged at 4 °C for 15 min at 13,600 × g. After discarding the supernatant, 750 μL of 75% ethanol was added for centrifugation at 16,000 × g for 10 min at 4 °C. The supernatant was discarded and centrifuged for 3 min under the same conditions. After ethanol volatilization, 10 μL DEPC water was added to dissolve the RNA. Finally, the concentration and purity of RNA were measured. The RNA solution was diluted to 62.5 ng/μL for reverse transcription. Reverse transcription reaction conditions were as follows: 37 °C, 15 min; 85 °C, 5 s. 20 μL of sterilized water was added to get the cDNA template and stored at 4 °C for later use. The reverse transcription reaction system consisted of the following:

**Table:** 

Reagent	Dose
5 × PrimeScriptTM RT Enzyme Mix I	2 μL
RNA fluid (62.5 ng/uL)	8 μL

### Real-time fluorescence quantitative PCR

mRNA sequences of required genes were retrieved from Genbank. Primer 6.0 software was used to design primers, and the best primer sequences were selected after comparison and sent to Guangzhou BGI Technology Co., LTD for synthesis. The primer series used are shown in Tables 1 and 2. Chama SYBR qPCR Master Mix kit was used in this test. According to the manufacturer’s instructions, the reaction system was set as follows:

**Table 1:** The primer sequences of the target gene

Gene	Primer sequence	Accession number	Product length
*Bax*	F:GGCCCTTTTGCTTCAGGGTT	XM_018062750.1	121
	R:CAGACACTCGCTCAGCTTCT		
*Bcl-2*	F:GAGTTCGGAGGGGTCATGTG	NM_001314213.1	152
	R:TACAGCTCCACAAAGGCGTC		
*β-actin*	F:CTCTTCCAGCCTTCCTTCCT	NM_001314342.1	177
	R:GGGCAGTGATCTCTTTCTGC

**Table 2:** The primer sequences of the target gene

Gene	Primer sequence	Accession number	Product length
*LC3B*	F:AGAAGGCGCTTACAGCTCAATGC	XM_018061829.1	92
	R:ACTTCACAAATCGGAGTGGACACAC		
*PINK1*	F: TCATCCAGCGAAGCCATCTTTAGC	XM_018055041.1	108
	R: TCCCTTGGGTCTTCCGTGAGTG		
*Parkin*	F:GCATAACGTGTACGGACATCAGGAG	XM_018053444.1	86
	R:CAGGTGGAAGCAGTCTAAGCAGATC		
*β-actin*	F:CTCTTCCAGCCTTCCTTCCT	NM_001314342.1	177
	R:GGGCAGTGATCTCTTTCTGC

**Table:** 

Reagent	Volume
2 × SYBR®primix Ex TaqTMII	5 μL
Upstream primer (4 μM)	1 μL
Downstream primer (4 μM)	1 μL
50 × ROX Reference Dye 1	0.2 uL
CDNA template	2.8 uL

The reaction conditions were pre-denaturation at 95 °C for 10 s, 40 cycles at 95 °C for 5 s, and annealing at 60 °C for 35 s. After the test, the CT values were derived for analysis. The relative expression differences among genes were analyzed by 2-∆ the CT method.

### Western blot analysis

Western blot was performed as previously described [37, 38]. Briefly, 50 mg of the small intestine samples were frozen at -80 °C, and 1 mL of lysate was added and homogenized on ice with a homogenizer. 500 μL tissue homogenate was transferred into a 1.5 mL centrifuge tube, and 1 mL of extraction reagent was added and mixed homogeneously. After allowing the mixture to stand at 4 °C for 10 min, it was centrifuged at 10,000 × g for 10 min at 4 °C; the solution was divided into upper and lower phases, the intermediate protein-membrane was retained, the upper and lower liquids were discarded, the tube mouth was opened, and the precipitation was dried at room temperature. An appropriate volume of 2% SDS solution was added to the mixture and boiled at 95 °C for 10 min, and allowed to stand at room temperature for 30 min. The protein supernatant was centrifuged at 12,000 RPM for 5 min. The protein concentration was determined by the BCA Kit (P1511, Applygen Technologies Inc., Beijing, China), and a protein loading buffer was added for backup. 8% gel was selected according to the molecular weight of the protein (30 ~ 100 kD). The proteins were separated by SDS polyacrylamide gel electrophoresis and blotted onto wet nitrocellulose membranes. PVDF membranes were sealed with 5% skim milk powder solution at room temperature for 2 h. After incubation with the primary antibody (diluted with 5% bovine serum albumin solution, with dilution ratios of Bax, Bcl-2 and β-actin of 1:1,000, 1:500 and 1:5,000, respectively). The primary antibodies used in this study include mouse anti-Bax antibody (ab77566, Abcam), mouse anti-Bcl-2 antibody (sc-7382, SAN cruz biotechnology (Shanghai) co., LTD and mouse anti-β-Actin antibody (BM0627, Boster, Wuhan, China). After overnight at 4 °C, the proteins were washed with TBST 3 times, for 10 min each time and incubated with secondary antibodies (Bax, Bcl-2 and β-actin diluted with 2% skim milk powder solution, with corresponding dilution ratios of 1:5,000, 1:2,000 and 1:10,000) at room temperature for 2 h, and washed with TBST 3 times. ECL luminescent solution was added for development. The experiment was repeated 3 times, an imaging system (Amersham Imager 600, General Electric, Boston, Massachusetts, USA) was used for the record of the ECL signals and the Image Pro Plus 6.0 software (Media Cybernetics, USA) was utilized for analysis. The relative expression levels were calculated using β-actin as the internal reference.

### Transmission electron microscope analysis

The sample was fixed with 2.5% glutaraldehyde and washed with PBS buffer 3 times. Then it was fixed with 1% osmium at 4 °C for 2 h and washed with PBS buffer 3 times for 10 min each time. The sample was dehydrated with graded ethanols (30%, 50%, 70%, 90%, and 100% ethanol) for 10 min and treated with 100% ethanol twice. The tissues were embedded with Epon812 epoxy resin and cured at 37 °C, 45 °C, and 65 °C. Tissue slices were obtained and stained with uranium dioxane acetate at room temperature for 30 min. The tissue slices were then cleaned and shaken dry with deionized water, stained with lead citrate for 8 min, washed with deionized water and dried. The results were observed and photographed under transmission electron microscopy.

### Statistical analysis

SPSS 23.0 software was used for one-way ANOVA analysis of the data obtained, and GraphPad Prism 8.0.2 software was used for mapping. The expression differences in genes and proteins at different transportation times were analyzed, and the data of each group were expressed as mean ± standard deviation. A the *P*-value < 0.05 was statistically significant, while a *P*-value < 0.01 was highly statistically significant.

### Supplementary Information

**Additional file 1.** Western Blot Original Images.

## Data Availability

The nucleotide sequences used in this study were collected from the National Center for Biotechnology Information (NCBI) GenBank repository. The GenBank accession numbers of all sequences were listed in Tables 1 and 2. The commercial antibodies used in this study are listed in the "Protein extraction and western blot analysis" section. The datasets used and/or analyzed during the current study are available from the corresponding author upon reasonable request.

## Abbreviations

ROS: Reactive oxygen species
SOD: Superoxide dismutase
MDA: Malondialdehyde
Bcl-2: B-cell lymphoma-2
Bax: BCL2-associated X
LC3B: Microtubule associated protein 1 light chain 3B
PINK1: PTEN induced putative kinase 1
CAT: Catalase
ECK: Extracellular regulated kinase
JNK: C-Jun N-terminal kinase
