# Supplementary material for: Effects of transport stress on the oxidative index, apoptosis and autophagy in the small intestine of caprine
Source: BMC Vet Res. 2023 Aug 9;19:117. doi: 10.1186/s12917-023-03670-9 (PMC10413633; doi:10.1186/s12917-023-03670-9)
Supplement: Supplementary file 1 — Additional file 1. Western Blot Original Images. [file 12917_2023_3670_MOESM1_ESM.zip › 1-Western Blot Original Images.docx]

**Western Blot Original Images**

We performed following experiments in article with cropped gels. All original images were showed as follows: “Time” represents the time of transport. This figure was showed the result of nine goats from three groups. This interpretation is also applied to the following all figures.

**In particular, the gels or impressions on some of the images were incomplete, this is due to some deviation in the position we placed during development, but this does not affect the integrity of the experiment.**

**
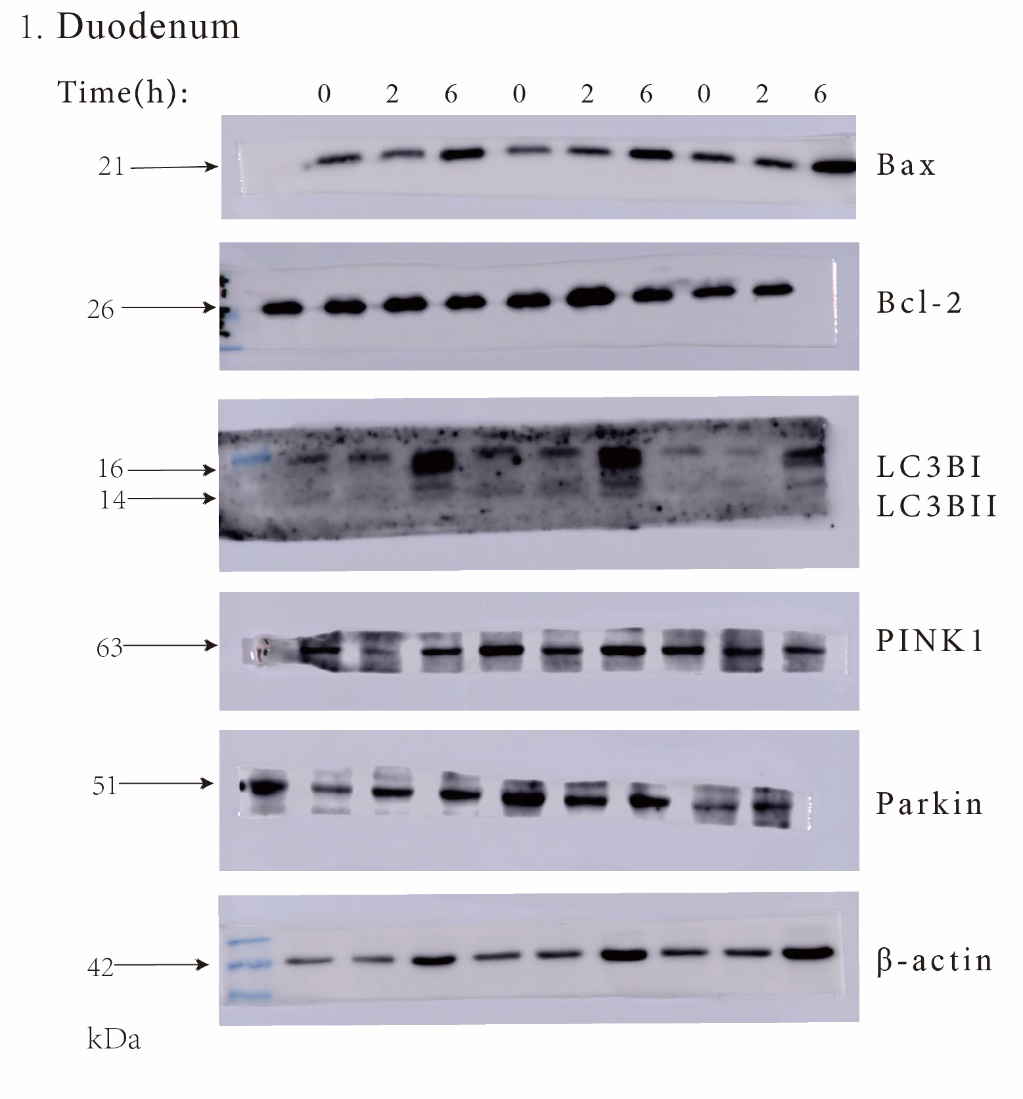
**

**
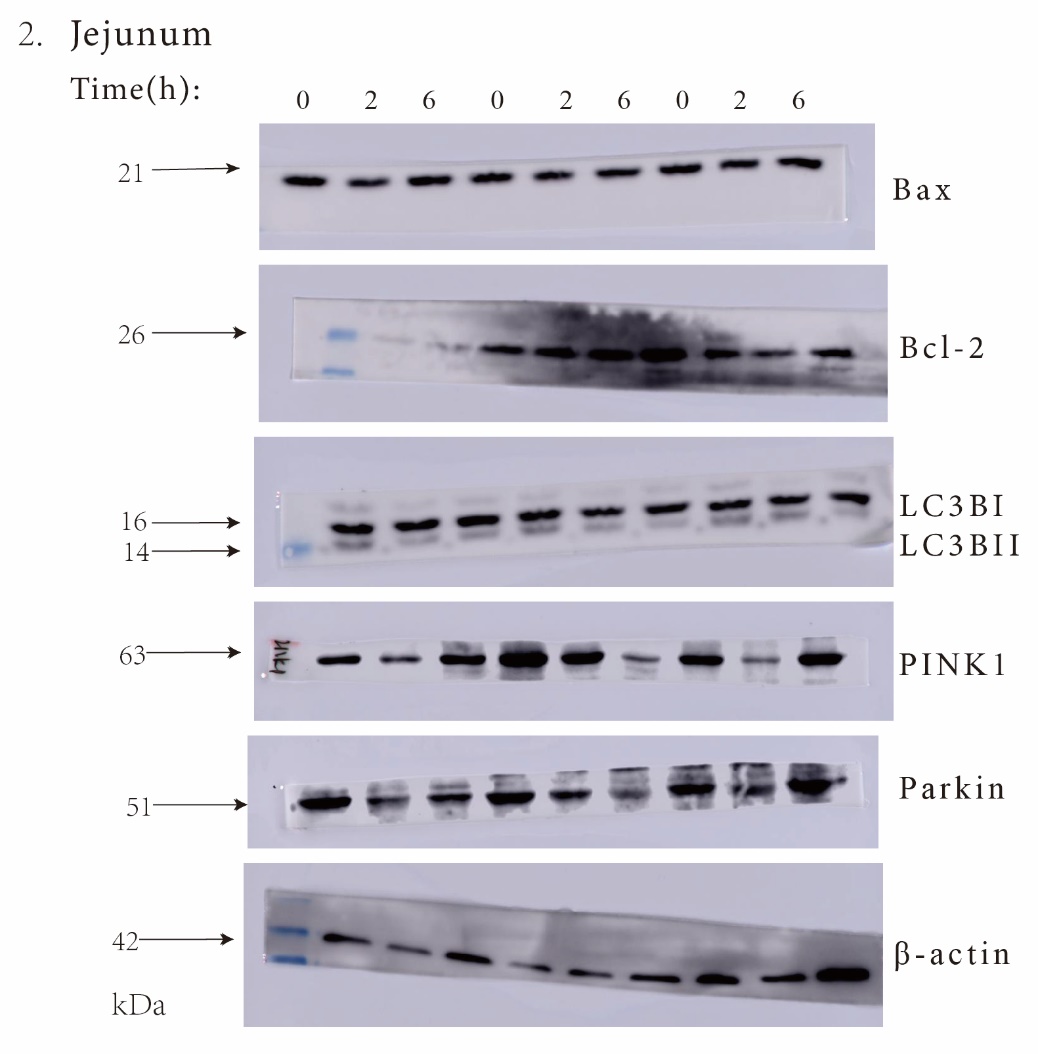
**

**
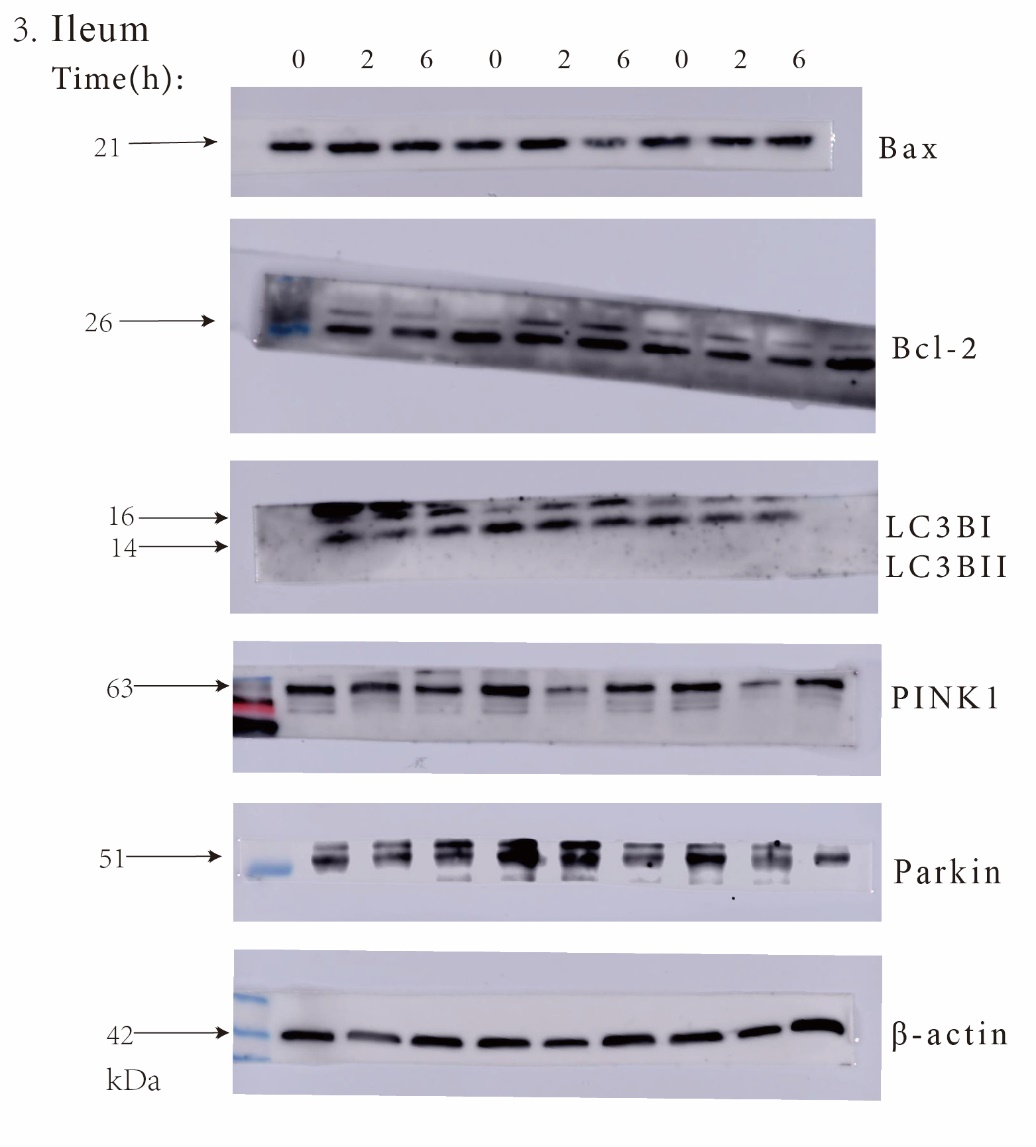
**
